# Reported Affect Changes as a Function of Response Delay: Findings From a Pooled Dataset of Nine Experience Sampling Studies

**DOI:** 10.3389/fpsyg.2021.580684

**Published:** 2021-02-26

**Authors:** Gudrun Eisele, Hugo Vachon, Inez Myin-Germeys, Wolfgang Viechtbauer

**Affiliations:** ^1^Department of Neurosciences, Center for Contextual Psychiatry, KU Leuven, Leuven, Belgium; ^2^Department of Psychiatry and Neuropsychology, School for Mental Health and Neuroscience, Maastricht University, Maastricht, Netherlands

**Keywords:** experience sampling, ecological momentary assessment, response delay, response latency, ambulatory assessment

## Abstract

Delayed responses are a common phenomenon in experience sampling studies. Yet no consensus exists on whether they should be excluded from the analysis or what the threshold for exclusion should be. Delayed responses could introduce bias, but previous investigations of systematic differences between delayed and timely responses have offered unclear results. To investigate differences as a function of delay, we conducted secondary analyses of nine paper and pencil based experience sampling studies including 1,528 individuals with different clinical statuses. In all participants, there were significant decreases in positive and increases in negative affect as a function of delay. In addition, delayed answers of participants without depression showed higher within-person variability and an initial strengthening in the relationships between contextual stress and affect. Participants with depression mostly showed the opposite pattern. Delayed responses seem qualitatively different from timely responses. Further research is needed to understand the mechanisms underlying these differences.

## Introduction

The experience sampling method (ESM; Csikszentmihalyi and Larson, 2014; Myin-Germeys et al., 2018) is a form of data collection that seeks to maximize ecological validity, reduce recall bias, and enable researchers to capture the dynamic nature of psychological phenomena in their context (Shiffman et al., 2008). This is achieved by prompting participants at (semi-)random or fixed times throughout the day to fill in a brief questionnaire about their feelings, thoughts, behaviors, and context. Despite the recent growth of ESM studies (Hamaker and Wichers, 2017; Van Berkel et al., 2018) and the increasing availability of comprehensive resources for researchers conducting them (e.g., Mehl and Conner, 2012; Bolger and Laurenceau, 2013), methodological research in this field remains scarce (Myin-Germeys et al., 2018). Consequently, evidence-based guidelines supporting the design of ESM studies are still lacking (Himmelstein et al., 2019). This is problematic not only because non-optimal methodological choices may introduce biases in the data collection but also because the resulting heterogeneity in ESM applications limits the comparability of results across studies (Tennen et al., 2006; Morren et al., 2009; Kockler et al., 2018; May et al., 2018).

One methodological aspect of the data collection procedure that has received little attention up to now is the allowed response delay (Janssens et al., 2018). The allowed response delay refers to the time after a prompt (“beep”) during which responses are still considered valid. When participants are prompted at unexpected or inconvenient moments, they are often not able or willing to respond immediately, which can lead to a delay in their responses (Stone and Shiffman, 2002). This response delay usually follows a positively skewed distribution, with most responses given within a couple of minutes (Csikszentmihalyi and Larson, 2014; Hofmann and Patel, 2015). Frequently, ESM researchers choose a maximal response delay, and responses that are given by participants later are either not recorded anymore or discarded before the analysis. The length of the allowed response delay varies, from seconds (Businelle et al., 2016) to hours after the prompt (Youngs and Graf, 2017; Janssens et al., 2018), with most studies allowing delays up to 30 min (Scollon et al., 2003). However, design choices such as the allowed response delay are often neither mentioned nor motivated in ESM papers (Morren et al., 2009).

Researchers that have addressed the issue have used different arguments for their choice of the allowed response delay. Practical considerations are often stated, such as giving participants enough time to respond (Cerin et al., 2001) or avoiding overlapping response windows (Janssens et al., 2018). Additionally, researchers have argued that delayed responses could introduce bias. Here, different types of ESM questions need to be distinguished that are potentially associated with different types of bias: Questions can either be momentary (i.e., concern the moment of filling in the questionnaire) or retrospective (i.e., ask participants to refer back either to the moment of the beep or the entire time period since the previous beep; Singh and Björling, 2019). In the case of momentary questions, the introduction of sampling or self-selection bias can be a concern when delayed responses are included in the analysis (Ainsworth et al., 2013). Sampling bias in this context refers to the issue that not all situations have an equal chance of being measured. While ESM researchers usually accept that certain situations will never be sampled (e.g., driving; Scollon et al., 2003), it is assumed that no systematic mood related bias is introduced (e.g., patients do not miss more signals when symptomatic; Ainsworth et al., 2013). Allowing participants to delay responses might lead to a violation of this assumption, for instance because participants wait for calm moments to fill in the questionnaire, thereby leading to a disproportionate high number of calm moments in the final sample. In the case of retrospective questions, delayed responses could introduce the recall bias that ESM studies originally sought to avoid (Scollon et al., 2003; Bolger and Laurenceau, 2013; Singh and Björling, 2019). Researchers have hypothesized that reports of mood would be most affected by this recall bias compared to, for instance, reports of activity or location (Delespaul, 1995). Since ESM is often treated as the standard against which the recall bias in other methods is compared (e.g., Kahneman et al., 2004), it is difficult to judge the presence of recall bias in delayed ESM data themselves. However, an indication of recall bias can be attained by investigating systematic differences between timely and delayed responses (Delespaul, 1995).

The few studies that examined the presence of systematic differences between delayed versus timely ESM responses have offered mixed results. No systematic differences were detected by MacKerron and Mourato (2013), who examined the link between environment (natural vs. urban) and momentary affect in a sample of more than 20,000 IPhone owners. Restricting the allowed response delay from up to 60 min to up to 20 min after the beep did not qualitatively change their results. Similar results were found by Affleck et al. (1998), who did check for systematic differences between timely (up to 5 min) and delayed (5–15 min) responses to retrospective items about pain, fatigue, and momentary items about mood. To this end, they randomly selected 100 timely and 100 delayed responses provided by 50 female fibromyalgia patients but did not find any significant differences between them. However, some studies did find that delayed responses were systematically different from responses given on time. Delespaul (1995), for instance, compared answers to retrospective items assessing affect, activity motivation, and psychotic symptoms given until 15 min after the beep to responses that fell outside of this time window in a sample of 167 individuals with different diagnoses of mental illness and healthy controls. He found indication of decreased variability in delayed responses, but no evidence for more social desirability. In a recent study, Sarker et al. (2014) found that in a sample of 30 university students higher reported happiness, being outside, and walking (when outside) were associated with a shorter response delay, while the response delay was longer in the mornings (at home), in the weekends, and while driving. In addition, participants were less likely to be available (i.e., able to respond within 124 s, irrespective of whether they answered later or not) when the GPS signal indicated that they were at work, walking at work, or driving. They were also less likely to be available when experiencing physiological stress (based on electrocardiograph and respiration sensors). In contrast, availability was found to be higher when participants were outside, walking (when outside), had just arrived home or were about to leave work, during weekends, and when feeling energetic or happy. However, the protocol employed differed in important ways from ESM protocols typically used in clinical populations (e.g., rewards were given for fast responding), which might limit the generalizability of the results. Along the same line, a recent investigation in 65 university students found that response delay was associated with the time of the day, day of the study, location, phone movement, and phone usage (Boukhechba et al., 2018). Previous research therefore indicates that some contextual characteristics may be associated with delay and that delayed responses of affect may be more positive and show less variance than responses given on time.

However, there is still insufficient evidence to draw conclusions about the presence of and reason for differences in delayed compared to timely responses, and as a result, the choice of the allowed response delay remains largely arbitrary (Scollon et al., 2003; Seekins et al., 2007). The objective of the current study was to work toward a better understanding of delayed responses in ESM data by investigating the presence of systematic differences as a function of delay in (a) mean levels of affect, (b) within-person variances of affect, and (c) associations between affect and contextual stress variables. Additionally, differences between individuals with different clinical status were explored. These research questions were investigated in a pooled dataset of nine ESM studies that followed similar protocols. All nine studies used paper-and-pencil-based ESM, which allowed participants to give delayed responses and therefore makes it possible to investigate changes as a function of delay.

## Materials and Methods

### Participants

The analyses were performed on a pooled dataset comprising nine paper-and-pencil ESM studies. Details about the individual studies can be found in Table 1. The studies included individuals suffering from depression, psychosis, at risk for psychosis (defined as being a first-degree relative of a patient with psychosis or being at high psychometric risk for psychosis), and control participants from the general population. The data that support the findings of this study are available from the corresponding author upon request.

**TABLE 1:** Overview of studies in the pooled dataset.

**Name**	**Sample (*n*) controls**	**Depressed**	**Psychotic**	**At risk**	**Sampling period in days**	Number of items**	**References**
Aripiprazole			27		4	51	Lataster et al., 2011b
Genetic Risk and Outcome of Psychosis (GROUP)*	85		72	81^*f*^	6	54	Collip et al., 2011; Lataster et al., 2013
Maastricht Psychosis Study (MAPS)	50		48	48^*f*^	6	50	Myin-Germeys et al., 2001
Mind Maastricht		129^1^			6	50	Geschwind et al., 2011
Stress-reactivity in Psychosis (STRIP)*	51		47	49^*f*^	6	55	Lataster et al., 2011a, 2014
PREVENT (Deutsch)	26		24	22^*p/f*^	6	55	Van der Steen et al., 2017
Mood and cortisol reactivity to daily stress*	39	45^2^			6	57	Peeters et al., 2006; Peeters et al., 2003
Twins*	610				5	50	Jacobs et al., 2007; Wichers et al., 2009
ZAPP	38		79	41^*p*^	6	53	Thewissen et al., 2008

*^∗^Study included saliva sampling. ^∗∗^The maximal number of questions including full branches. ^*p*^Psychometric risk (defined as subclinical symptoms detected with questionnaires or structured interviews). ^*f*^Familial risk. ^1^Lifetime history of depression and current residual symptoms. ^2^Current score ≥ 18 on Hamilton depression rating scale.*

### Experience Sampling Method Protocol

At the beginning of all included studies, participants received a wristwatch and a paper booklet containing the questionnaires. The wristwatch delivered 10 beeps per day at random moments within 10 90-min windows from 7:30 until 22:30. Participants had not been informed about the length of the response window that would be applied to the data but were instructed to always carry wristwatch and booklet with them and respond as quickly as possible after each beep. They were also instructed to refer back to the moment of the beep when they answered the questions and to report the time when they started filling in the questions. Four of the included studies used saliva sampling alongside ESM (see Table 1). In some of the studies, participants who responded to less than one third of the scheduled beeps had been irreversibly deleted from the dataset, in line with common practice (Delespaul, 1995). To homogenize the data, the same exclusion criterion was applied to the whole dataset. Incentives differed per studies but did not depend on the compliance to the beeps to avoid encouraging backfilling.

### Measures

#### Affect and Contextual Stress Variables

Items that were assessed in all studies of the pooled dataset were used in the current analysis. The average of the two items “I feel cheerful” and “I feel satisfied” was used to assess the level of positive affect at each beep (reliability calculated according to Shrout and Lane (2012), *K*_*c*_ = 0.81 and *K*_*kf*_ = 0.99). The average of the five items “I feel lonely/anxious/irritated/down/guilty” was used to assess negative affect (*K*_*c*_ = 0.62 and *K*_*kf*_ = 0.99). Stress related to the current activity a participant was engaged in at the time of the beep (“activity stress”) was assessed by calculating the average of four items that asked participants to rate their current activity (“I prefer doing something else,” “This activity costs energy,” “I am skilled” – reverse coded, “This is a challenge”; *K*_*c*_ = 0.46 and *K*_*kf*_ = 0.95). Answers for affect and activity stress items were given on Likert scales ranging from 1 to 7, and the numbers 1, 4, and 7 were marked with the labels “not at all,” “moderately,” and “very much,” respectively. Stress related to important events (“event stress”) was assessed with one item that asked participants to rate the valence of the most important event that had occurred since the last beep on a bipolar scale ranging from −3 (“This event was very unpleasant”) to +3 (“This event was very pleasant”). For consistency with the other items, this variable was recoded to a 1 to 7 scale (i.e., −3 to 7, −2 to 6, …, +3 to 1) so that a higher score indicated a more negative valence.

#### Delay

**FIGURE 1:**
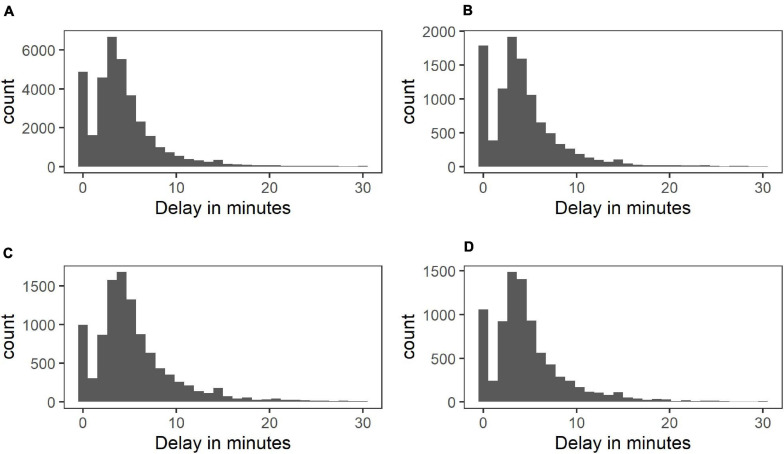
Distribution of response delays in control **(A)**, at-risk **(B)**, psychotic **(C)**, and depressed group **(D)**.

The measure of delay was calculated by subtracting the scheduled time of a beep (as programmed into the wrist watch) from the participant’s reported time of filling in the questionnaire (in minutes). Negative delays were included until up to 5 min before the beep and set to 0 in the analyses. Negative delays are common in paper and pencil ESM data (Delespaul, 1995). They can be caused for instance by time discrepancies between the experimental device and the clock that the participant is referring to for noting the time or by backfilling. This topic is further addressed in the discussion section. Analyses were repeated excluding responses with negative delays and discrepancies are described in the results section. Positive delays up to 30 min were included in the analyses because very few responses were given after 30 min in the current sample (see Figure 1 for the distribution of included delays).

#### Time

Study day number (ranging from 0 to 5) was included as a measure of time in the analyses to account for overall changes in outcomes over the course of the studies. To control for possible confounding of diurnal patterns in affect and delay, the number of beeps within each day (ranging from 0 to 9) was also included in the analyses.

#### Clinical Status

The clinical status of the study participants was coded as a 4-level factor (with levels: control, at-risk for psychosis, diagnosed with psychosis, and diagnosed with depression), with the control participants as the reference category.

### Analysis

To account for the hierarchical structure of the data, several multilevel regression models were fitted with occasions/beeps at level one nested within persons at level two. Because of the unknown and possibly nonlinear functional form of the relationship between delay and affect and the previously observed nonlinear diurnal pattern of positive affect ratings (e.g., Murray et al., 2002), restricted cubic splines (Stone and Koo, 1985) were used for beep number and delay, as they allow flexible modeling of nonlinear relationships and offer a better fit than simple polynomials in many cases (Harrell, 2015). The locations of the knot points for the splines were based on the percentiles of the predictor variables to ensure sufficient data points in each interval and hence stable estimates (Harrell, 2015). To balance flexibility with the need to avoid overfitting the data with too many parameters, three knots were considered appropriate because of the numerous other predictors in the models and were set for delay and beep number separately. Following Harrell (2015), the position of the three knots were based on the 10th, 50th, and 90th percentiles, resulting in the positions 1.9, 5.5, and 9.1 for beep number. Since the 10th percentile for response delay was 0, we instead used the recommended percentiles for four knots (i.e., the 5th, 35th, 65th, and 95th percentile), leaving out the first knot (because there is no data below 0 that can be used to estimate changes in the trajectory at this knot location), resulting in knot positions of 3, 5, and 12 min. To assess the significance of fixed effects, Wald-type tests were conducted. Because delay was represented by two separate variables (i.e., the original variable and a transformed variable based on the cubic spline), the significance of delay as a fixed predictor was assessed by testing both terms simultaneously using a chi-square Wald-type test with two degrees of freedom.

Three types of analyses were conducted. The first examined if there is an association between the response delay and the level of positive and negative affect reported by participants (i.e., do participants report higher or lower levels of PA/NA when responding to a beep with increasing delay?). In the second analysis, we examined if the amount of within-person variability differed as a function of the response delay (i.e., do participants’ responses fluctuate more or less when responding to a beep with increasing delay?). Finally, we examined if the association between each of the two contextual stress variables (i.e., activity and event stress) and affect differed as a function of the response delay (i.e., is the association between stress and affect stronger or weaker when responding to a beep with increasing delay?). We now describe the corresponding models in more detail.

(a) Changes in levels of affect as a function of delay

Two models were fitted to predict PA and NA from delay. The models included either PA or NA as an outcome variable and delay, day number, beep number, clinical status, and the interaction between clinical status and delay as predictors. In addition to random intercepts, random slopes were included for delay (both terms) and beep number (both terms). Adding a random slope for day number led to convergence problems and was subsequently left out in all analyses. An unstructured variance–covariance matrix was used for the random effects. Omnibus tests were conducted to check if there were group differences in the association between affect and delay (all groups compared to the control group). Significant omnibus tests were followed by pairwise comparisons to investigate which groups differed significantly from the control group.

(b) Changes in within-person variances of affect as a function of delay

The two models used to address the first question were extended by modeling heterogeneity in the within-person variance component as a function of delay and clinical status. In particular, instead of adopting the usual homoscedasticity assumption that the within-person variance, σ^2^, does not depend on any characteristics of the measurements, we used a location-scale multilevel model (Hedeker et al., 2012; implemented in the nlme package in R; Pinheiro et al., 2018), where the within-person variance for a particular participant *i* at a particular beep *j* is given by

$$\sigma_{i\,j}^{2}=\sigma^{2}\,e\,x\,p\,\left(\alpha_{2}\,G_{2\,i}+\alpha_{3}\,G_{3\,i}+\alpha_{4}\,G_{4\,i}+\beta_{1}\,D_{i\,j}+\beta_{2}\,D_{i\,j}\,G_{2\,i}+\beta_{3}\,D_{i\,j}\,G_{3\,i}+\beta_{4}\,D_{i\,j}\,G_{4\,i}\right)$$

where *G*_*2i*_, *G*_*3i*_, and *G*_*4i*_ are dummy variables to indicate membership in groups 2 (at risk for psychosis), 3 (psychosis), and 4 (depressed) and *D*_*ij*_ is the response delay for a particular participant at a particular beep. Hence, σ^2^ is the within-person variance in group 1 (controls) when the delay is 0; exp(α_2_), exp(α_3_), and exp(α_4_) are multiplicative factors that allow the within-person variances for the other groups to differ from that of the control group (e.g., σ^2^exp(α_2_) is the variance for group 2); exp(β_1_) is a multiplicative factor that indicates how the within-person variance of the control group changes per 1-min increase in delay (e.g., σ^2^exp(β_1_×5) is the variance of the control group for a 5-min delay); and exp(β_2_), exp(β_3_), and exp(β_4_) are multiplicative factors that indicate how much more or less the within-person variance of the other groups changes per 1-min increase in delay compared to the control group (e.g., σ^2^exp(α_2_ + (β_1_ + β_2_)×5) is the variance for group 2 for a 5-min delay).

We tested *H*_0_:β_1_ = 0 to examine if the within-person variance changes as a function of delay in the control group. Subsequently, we tested *H*_0_:β_2_ = 0, *H*_0_:β_3_ = 0, and *H*_0_:β_4_ = 0 to assess if the other groups differed from the control group in the rate of change of the within-person variance as a function of delay.

(c) Changes in associations between affect and contextual stress variables as a function of delay

Separate models for PA and NA were fitted for each of the two contextual stress variables (i.e., activity and event stress). The models included the main effects plus two and three-way interactions of delay, clinical status, and the contextual stress variable. Beep number and day number were included as control variables. Random effects were added as in the previous models.

All predictor variables were rescaled prior to the analysis to facilitate convergence, but untransformed coefficients are depicted in the graphs for the sake of clarity. To correct for multiple testing, we applied a Bonferroni correction while taking into account all tests of interest performed (0.05/29 = 0.0017). Analyses were performed in R version 3.4.3 using the nlme (version 3.1-131; Pinheiro et al., 2018), rms (version 5.1-2; Harrell, 2018), and car (version 2.1-6; Fox and Weisberg, 2011) packages and SAS 9.4 using proc mixed (due to the complexity of the models, results were cross-validated across software). The code for the analyses can be found in the Supplementary Material.

## Results

Sixty participants were excluded from the analyses because they responded to less than one third of the beeps, leaving 1,528 participants (see Supplementary Table 1 for sample characteristics). The median response delay was 3 min for control participants (IQR = 3) and 4 min for at-risk participants (IQR = 3), as well as for the participants with a diagnosis of psychosis and depression (IQR = 4; see Figure 1). The distribution of delays in Figure 1 indicates that a high number of answers given at the moment of the beep were followed by the typical, skewed distribution of delays. Due to missing responses on some predictor variables, the sample sizes varied per analysis. The number of participants included in each analysis is stated in Supplementary Tables 2–9, which contain the full results of all fitted regression models.

(a) Changes in mean levels of affect as a function of delay

**FIGURE 2:**
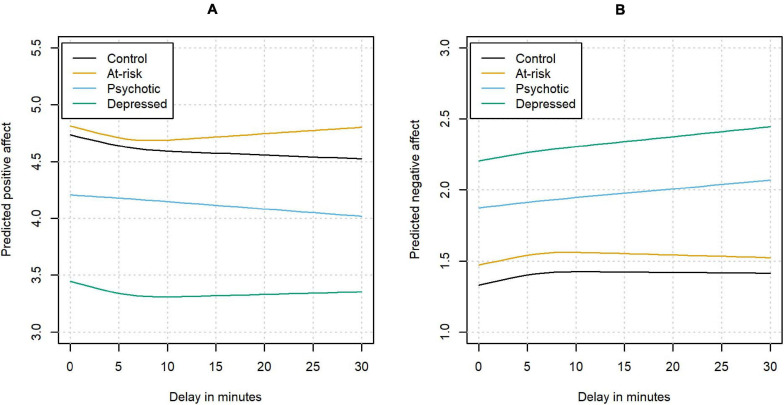
The association between delay and positive affect **(A)** and negative affect **(B)**.

We found subtle but systematic changes in both PA and NA as a function of response delay. The results are depicted in Figure 2 in terms of the predicted mean PA as a function of the response delay. For PA, a significant association with delay could be detected in the control group (χ^2^ (2) = 30.69, *p* < 0.001). The association was not significantly different for different status groups (χ^2^ (6) = 5.65, *p* = 0.46), which all showed an initial decrease of PA with longer delays.

A significant association with delay could also be detected for NA in the control group (χ^2^ (2) = 37.72, *p* < 0.001). Again, this effect was not significantly different in the other status groups (χ^2^ (6) = 9.27, *p* = 0.16), which all showed an increase in NA with longer delays.

(b) Changes in within-person variances of affect as a function of delay

**FIGURE 3:**
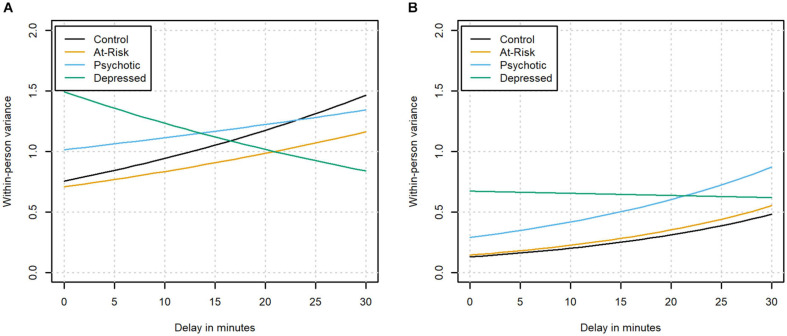
The within-person variance of positive **(A)** and negative affect **(B)** as a function of the delay in the different clinical groups.

Next, we investigated possible changes in the within-person variance of affect ratings as a function of the delay. The within-person variance corresponds to the variance in affect ratings that is not explained by other predictors in the regression model. Instead of assuming that this variance is independent of all predictors in the model, the location-scale model relaxes this assumption and allows the within-person variance to change as a function of the delay. This means that the model estimates both the within-person variance at the moment of the beep and the increase in within-person variance with every unit increase in delay for individuals in each of the clinical status groups. The resulting estimates can be found in Figure 3 and Supplementary Tables 4, 5. In the absence of a difference between timely and delayed responses, we would not expect significant changes in the within-person variance of affect ratings as a function of the delay in any of the groups. The within-person variances of PA and NA changed systematically with longer delays, and this effect was moderated by clinical status (see Figure 3 for a graphical depiction). For positive affect, the variance in the control group was found to increase significantly as a function of response delay (*z* = 10.71, *p* < 0.001). An increase in the variance could also be observed in the participants at-risk and with a diagnosis of psychosis. While the increase did not differ from the control group in the at-risk group (*z* = −1.28, *p* = 0.20), the increase in the group with a diagnosis of psychosis was significantly smaller than the increase found in the control group (*z* = −3.48, *p* < 0.001). On the other hand, the variance in the group diagnosed with depression was found to decrease with longer delays, which represented a significant difference from the control group (*z* = −10.01, *p* < 0.001).

For negative affect, the variance also increased significantly as a function of response delay in the control group (*z* = 19.79, *p* < 0.001). This effect was not significantly different in the group identified as being at-risk or the group diagnosed with psychosis in which the variances were also found to increase (*z* = 0.28, *p* = 0.78 and *z* = −1.75, *p* = 0.08, respectively). In the group diagnosed with depression, the variance remained stable with longer delays, which represented a significant difference from the control group (*z* = −11.44, *p* < 0.001).

(c) Changes in associations between affect and contextual stress as a function of delay

Finally, changes in the association between contextual stress and affect as a function of the delay were modeled in every clinical status group. Specifically, we tested the significance of the interaction between each contextual stress variable (i.e., activity stress and event stress) and delay in models predicting affect. For simplicity, the effect of delay on the within-person variance of affect was excluded from these models. The interaction between contextual stress and delay was further allowed to interact with the clinical status variable, meaning that the interaction could differ depending on the clinical status of a participant. In the absence of a difference between timely and delayed responses, we would not expect the interaction between contextual stress and delay to be significantly different from 0 in any of the groups.

**FIGURE 4:**
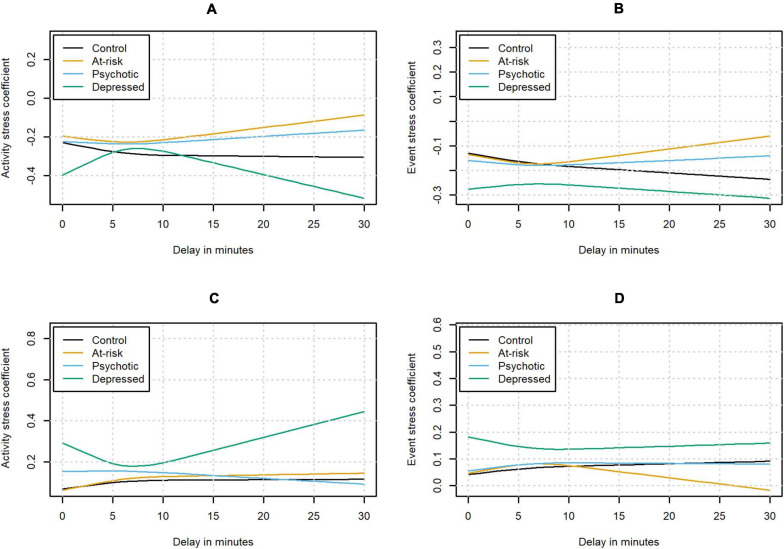
Estimated contextual stress coefficients for the relationship between PA and activity stress **(A)** and event stress **(B)**, and for the relationship between NA and activity stress **(C)** and event stress **(D)** as a function of the delay.

The associations between the contextual variables and affect were found to change with longer delays. However, the changes were inconsistent across status groups and contextual variables. The interactions (in terms of the estimated model coefficient for the relationship between affect and the contextual stress variable as a function of the delay) are shown in Figure 4. Activity stress was negatively associated with PA at the moment of the beep, meaning that when participants reported higher activity stress, they tended to report lower PA. This negative association between PA and activity stress was found to become more negative (i.e., stronger) for longer delays in the control group, but the interaction (between delay and activity stress) did not meet the Bonferroni-corrected threshold of significance (χ^2^ (2) = 9.62, *p* = 0.008). At the same time, the omnibus chi-square test indicated a significant three-way interaction of activity stress with delay and status (χ^2^ (6) = 22.10, *p* = 0.001). This effect was not significant anymore when excluding negative delays. This interaction was primarily driven by the group diagnosed with depression, which showed a pattern significantly different from the one in the control group (χ^2^ (2) = 17.24, *p* < 0.001); here the association between activity stress and PA initially became less negative with longer delays before becoming more negative again. On the other hand, the interaction effect was not significantly different from the control group in the group identified as being at-risk and the group diagnosed with psychosis (χ^2^ (2) = 3.64, *p* = 0.16; χ^2^ (2) = 3.36, *p* = 0.19).

Event stress was negatively associated with PA at the moment of the beep. A significant interaction of event stress and delay was found in the control group (χ^2^ (2) = 16.53, *p* < 0.001), with the negative association between event stress and PA becoming more negative with longer delay. The omnibus test did not indicate any significant differences in the clinical status groups compared to the control group (χ^2^ (6) = 12.48, *p* = 0.05).

Activity stress was positively associated with NA at the moment of the beep. A significant interaction between delay and activity stress was found in the control group (χ^2^ (2) = 14.11, *p* < 0.001) with the positive association between activity stress and NA initially becoming more positive with longer delays. After the initial increase, the change in the association between activity stress and NA flattened out. Additionally, the omnibus chi-square test indicated a significant three-way interaction of activity stress with delay and status (χ^2^ (6) = 49.99, *p* < 0.001). Pairwise comparisons indicated that the effect was not significantly different in the participants who were at-risk or had already developed a psychosis compared to the control group (χ^2^ (2) = 1.12, *p* = 0.57 and χ^2^ (2) = 7.44, *p* = 0.02, respectively). On the other hand, the group diagnosed with depression again showed a significantly different pattern from the control group with the association between activity stress and NA initially becoming less positive (χ^2^ (2) = 32.27, *p* < 0.001). For delays longer than approximately 7 min, the association between activity stress and NA was becoming more positive again.

A similar pattern could be observed when predicting NA from event stress. Event stress was positively associated with NA at the moment of the beep. In the control group, a significant interaction of event stress and delay was observed, with the association between event stress and NA initially becoming more positive with longer delays (χ^2^ (2) = 15.38, *p* < 0.001). A significant three-way interaction of event stress with delay and status could be detected (χ^2^ (6) = 28.81, *p* < 0.001). In the participants at-risk or with a diagnosis of psychosis, the interaction was not significantly different from the control group after controlling for multiple testing (χ^2^ (2) = 0.60, *p* = 0.74; χ^2^ (2) = 8.15, *p* = 0.02). As before, the group with depression showed a significantly different pattern from the control group with the association between event stress and NA initially becoming less positive with longer delays (χ^2^ (2) = 18.70, *p* < 0.001). With even longer delays, the association was found to become more positive again.

## Discussion

A main advantage of ESM over retrospective questionnaires is the collection of data in real time. We found that in our sample, responses were often given with a 3- to 4-min delay after the original signal. The current study was the first to investigate the consequences of such delayed responses in different clinical populations. Systematic differences between timely and delayed responses could be detected in the (a) mean and (b) variance of affect and (c) in the associations between affect and contextual stress. In addition, significant heterogeneity in these differences was found between individuals with and without a diagnosis of depression.

We found significant changes in reported affect as a function of delay. In all groups, PA decreased and NA increased the longer the time difference between the beep and filling in of the questionnaire became. Effect sizes for the difference in predicted PA between a delay of 0 and 10 min were small, ranging from −0.06 in the group with psychosis to −0.17 in the control group. For changes in predicted NA immediately vs. 10 min after the beep, the effect sizes ranged from 0.13 in the participants with depression and psychosis to 0.26 in the control group. Effect sizes were calculated per clinical status group with the following formula: ES = (*M*_2_−*M*_1_)/σ_*ij*_, with *M_1* denoting the predicted affect at the moment of the beep, *M_2* the predicted affect after 10 min of delay for this group, and $\sigma_{i\,j}^{2}$ the estimated within-subject variance for a subject *i* of a specific group at beep *j* with delay 0, calculated based on the estimates from question 2 (see Supplementary Tables 3, 4). This finding is in line with previous investigations showing a positive association between positive affect and shorter response delays in an ESM study (Sarker et al., 2014).

While the available data do not allow us to rule out any of the possible explanations with certainty, we would like to discuss several possible mechanisms that may explain these changes. The observed decrease in PA and increase in NA could either be due to actually experienced changes in affect or increased measurement error as a function of delay. If they are due to real changes in affect, one possible explanation is that situational constraints that lead to longer delays are also associated with lower PA and higher NA. An example could be a participant feeling anxious while engaging in a stressful activity and delaying responding as a result. In line with this reasoning, previous studies showed the predictive value of objectively measured contextual characteristics (such as location and activity) on response delay (Sarker et al., 2014; Boukhechba et al., 2018). Further, physiologically measured stress was previously found to relate to the availability to respond to a beep (Sarker et al., 2014). On the other hand, the decreased PA and heightened NA with longer delays may also be caused by burden induced by the protocol (Fuller-Tyszkiewicz et al., 2013). Increased disturbance of the beep in situations that do not allow immediate responding could lead to both delay and to changes in the emotional experience of participants (i.e., reactivity; Hektner et al., 2007). Based on the current data, it is not possible to distinguish if the negative affect was caused by the beep or already present before the beep occurred.

Beyond these options, response delays could also be an indication that participants are engaged in another activity or not motivated to respond, both of which could imply that they also pay less attention to the responses they give. In line with this, a recent qualitative report found reduced self-reported data accuracy when participants were multitasking (Van Berkel et al., 2019). The intensity of ESM assessments may thereby induce careless responding (Van Berkel et al., 2018), which could lead to seeming changes in mean affect. Specifically, carelessly given responses may be random (Fuller-Tyszkiewicz et al., 2013), tend to default values, toward the middle, or one of the endpoints of the scale (i.e., midpoint, acquiescent, or disacquiescent response style; Stone et al., 2019), all of which could lead to changes in affect. Finally, another form of measurement error, namely, recall bias, could also explain the changes in affect. In this case, the changes would be a direct result of the response delay because participants have trouble remembering their affect at the beep (Scollon et al., 2003; Takarangi et al., 2006). This inability to remember could lead to systematic biases (Ben-Zeev et al., 2009).

The second and third results allowed us to investigate the changes in affect as a function of delay in more detail. In individuals without depression, we observed that the variance of PA and NA ratings increased with longer delays. Furthermore, the relationships between contextual stress and affect initially became stronger with longer delays in participants without depression (except for the relationship between activity stress and PA, where the positive interaction with delay was not significant). When participants answer carelessly, they may answer randomly or, on the contrary, very uniformly (Fuller-Tyszkiewicz et al., 2013). In the first case, the careless responding could lead to an increase in within-person variance as observed in the participants without diagnosed depression. However, if participants had answered truly randomly, we would also expect the relationships between context and affect to become weaker, which was not the case in the group without depression. In the case of prototypical responding on the other hand, we would not expect the increase in variance found in these participants. This may suggest that the changes in the groups without depression were not due to careless responding.

Remarkably, the group with a diagnosis of depression showed different changes as a function of delay compared to the other investigated samples. In contrast to the participants without depression, they showed lower within-person variance for PA with longer delays and no decrease of the variance of NA ratings. At the same time, the relationships between contextual stress and affect initially became weaker in delayed responses in participants with depression, as opposed to the strengthening in participants without depression (except for the relationship between PA and event stress, where we found no significant difference with the control group). This suggests that a different mechanism may underlie the changes in PA and NA in individuals with depression. It needs to be noted that 3 of the 4 relationships between contextual stress and affect showed a strengthening after the initial weakening in the group with diagnosed depression, which might indicate that the process is qualitatively different for responses given after more than 7 min. However, less data was available to estimate the effect of these longer delays, which means that the observed changes need to be interpreted with caution.

## Limitations and Future Research

A number of limitations should be taken into account when interpreting the results. The current analyses were conducted with data collected using paper-and-pencil assessment, which has been associated with backfilling in the past when fixed time sampling schemes were used (e.g., Stone et al., 2002). However, the current investigation was conducted with ESM data that were collected based on a semi-random sampling scheme, which may make it more difficult for participants to accurately backfill the time of the beep (Green et al., 2006). Additionally, several measures were taken in the included studies to reduce participants’ motivation to backfill responses. For instance, the payment of participants was not dependent on their compliance and participants were actively encouraged during the briefing to stick to their daily routines and activities even if that would mean missing beeps. The times when participants reacted to the beeps were also checked unobtrusively in a subsample of one of the included studies and high accordance of these times with scheduled beep times was recorded (Jacobs et al., 2005; De Wild-Hartmann et al., 2013). However, the prevalence of negative response delays suggests that there may still have been cases of backfilling. We would expect that this only applies to a small percentage of the given responses and would not systematically affect the results, but we cannot rule out this possibility. A related potential limitation is that processes underlying response delays may be different in electronically assessed ESM data. Therefore, both the possible distortion by backfilling and the applicability of the current results to electronic assessments should be investigated in the future. Another limitation is that group differences could have been distorted because the groups were relatively heterogeneous (e.g., some lifetime diagnosis, others currently ill, etc.). Additionally, the internal consistency/reliability of the within-person change of the activity stress ratings was below traditional thresholds for acceptable reliability. This indicates that the items are measuring distinct forms of activity stress that did not necessarily co-occur in participants’ daily lives. However, the average of these distinct forms of stress can still be informative, as situations leading to high scores on all included forms of activity stress are arguably more stressful than situations that are associated with only one form of stress. Further research is needed to clarify the reasons for the observed changes and differences based on clinical status. The effect of recall bias could for instance be investigated by manipulating the delay between beep and response. Data-driven interviews and use of smartphone sensors could also be used to gather more information about the context surrounding beeps when responses are delayed.

## Conclusion

Systematic differences between timely and delayed responses could be detected in a large sample of nine pooled ESM studies including participants with varying clinical status. Delayed responses were found to show higher NA and lower PA. Additionally, differences in the within-person variance of affect and the relationships between affect and contextual stress could be detected with longer delays, with the group with depression showing a significantly different pattern than the control group. While the reasons for the observed differences remain unclear, the current findings indicate that there are qualitative differences in responses given after increasing delays. Further research is needed to explore the exact mechanisms behind these changes and to work toward setting a reasonable guideline that can help to homogenize the way that delayed ESM responses are handled.

## Data Availability Statement

The data analyzed in this study is subject to the following licenses/restrictions: The data are not publicly available, but can be shared upon request. Requests to access these datasets should be directed to IM-G, inez.germeys@kuleuven.be.

## Ethics Statement

These datasets were collected for 9 individual studies that were each reviewed and approved individually by the Medical Ethical Committee at Maastricht University (Netherlands; Medisch Ethische commissie azM/UM). The patients/participants provided their written informed consent to participate in the studies.

## Author Contributions

GE contributed to conceptualization, methodology, formal analysis, investigation, writing, and visualization. HV contributed to conceptualization, methodology, writing – review and editing, and supervision. IM-G contributed to conceptualization, writing – review and editing, supervision, and funding acquisition. WV contributed to conceptualization, methodology, software, writing – review and editing, and supervision. All authors contributed to the article and approved the submitted version.

## Conflict of Interest

The authors declare that the research was conducted in the absence of any commercial or financial relationships that could be construed as a potential conflict of interest.
