# Supplementary material for: Reported Affect Changes as a Function of Response Delay: Findings From a Pooled Dataset of Nine Experience Sampling Studies
Source: Front Psychol. 2021 Feb 26;12:580684. doi: 10.3389/fpsyg.2021.580684 (PMC7952513; doi:10.3389/fpsyg.2021.580684)
Supplement: Supplementary file 1 [file Data_Sheet_1.docx]

Supplementary material

| Table S1. *Sample characteristics.* | | | | |
| --- | --- | --- | --- | --- |
| Clinical status | Control | At-Risk | Psychotic | Depressed |
| N | 873 | 229 | 252 | 174 |
| Female (%) | 83.4 | 53.7 | 32.6 | 71.3 |
| Age  *M (SD)* | 31.3 (10.9) | 36.8 (13.1) | 34.5 (11.0) | 42.8 (10.1) |
| Compliance (%) | 72.4 | 76.5 | 69.5 | 80.7 |
| Response delay *Median* (*IQR*) | 3 (3) | 4 (3) | 4 (4) | 4 (4) |
| PA  *M (SD)* | 4.85 (1.28) | 4.95 (1.32) | 4.37 (1.45) | 3.52 (1.60) |
| NA  *M (SD)* | 1.29 (0.58) | 1.41 (0.74) | 1.81 (1.03) | 2.17 (1.16) |
| Event stress  *M (SD)* | 2.73 (0.16) | 2.62 (0.16) | 2.70 (0.17) | 2.75 (0.18) |
| Activity stress  *M (SD)* | 2.45 (0.10) | 2.43 (0.11) | 2.67 (0.11) | 2.69 (0.11) |
|  | | | | |

| Table S2. *The association between delay and positive affect. Coefficients for rescaled variables.* | | | | | |
| --- | --- | --- | --- | --- | --- |
| Fixed Effects | β | *SE* | *DF* | *t* | *p* |
| Intercept | 4.6359 | 0.03421 | 1523 | 135.53 | <.0001 |
| Delay | -0.1978 | 0.04762 | 1518 | -4.15 | <.0001 |
| Delay_cs | 0.2463 | 0.09905 | 1492 | 2.49 | 0.0130 |
| Status 1 | 0.06948 | 0.06953 | 57000 | 1.00 | 0.3177 |
| Status 2 | -0.4572 | 0.06667 | 57000 | -6.86 | <.0001 |
| Status 3 | -1.2989 | 0.07723 | 57000 | -16.82 | <.0001 |
| Delay* Status 1 | -0.01866 | 0.09766 | 57000 | -0.19 | 0.8485 |
| Delay* Status 2 | 0.1401 | 0.09999 | 57000 | 1.40 | 0.1612 |
| Delay* Status 3 | -0.02152 | 0.1103 | 57000 | -0.20 | 0.8453 |
| Delay_cs* Status 1 | 0.1637 | 0.2049 | 57000 | 0.80 | 0.4244 |
| Delay_cs* Status 2 | -0.2563 | 0.1996 | 57000 | -1.28 | 0.1991 |
| Delay_cs* Status 3 | 0.1167 | 0.2264 | 57000 | 0.52 | 0.6062 |
| Beep number | 0.6744 | 0.04770 | 1526 | 14.14 | <.0001 |
| Beep number_cs | -0.6307 | 0.05523 | 1527 | -11.42 | <.0001 |
| Day number | -0.09864 | 0.02520 | 57000 | -3.91 | <.0001 |
| Random effects | β | *SE* | *Z* | *p* |  |
| Intercept | 0.9285 | 0.04616 | 20.12 | <.0001 |  |
| Delay | 0.4577 | 0.06559 | 6.98 | <.0001 |  |
| Delay_cs | 1.5913 | 0.2691 | 5.91 | <.0001 |  |
| Beep number | 1.1295 | 0.1258 | 8.98 | <.0001 |  |
| Beep number_cs | 1.2781 | 0.1691 | 7.56 | <.0001 |  |
| Residual | 0.9831 | 0.005786 | 169.91 | <.0001 |  |
| *Notes. N* = 1528; Number of Observations = 64110; Delay_cs = cubic spline transformed delay; Beep number_cs = cubic spline transformed beep number; Reference category = control, status 1 = individuals at risk for psychosis, status 2 = individuals with psychosis, status 3 = individuals with depression. | | | | | |

| Table S3. *The association between delay and negative affect. Coefficients for rescaled variables.* | | | | | |
| --- | --- | --- | --- | --- | --- |
| Fixed Effects | β | *SE* | *DF* | *t* | *p* |
| Intercept | 1.4031 | 0.02099 | 1522 | 66.85 | <.0001 |
| Delay | 0.1427 | 0.02487 | 1517 | 5.74 | <.0001 |
| Delay_cs | -0.2223 | 0.05230 | 1491 | -4.25 | <.0001 |
| Status 1 | 0.1406 | 0.04342 | 56000 | 3.24 | 0.0012 |
| Status 2 | 0.5106 | 0.04167 | 56000 | 12.25 | <.0001 |
| Status 3 | 0.8607 | 0.04831 | 56000 | 17.81 | <.0001 |
| Delay* Status 1 | 0.001630 | 0.05066 | 56000 | 0.03 | 0.9743 |
| Delay* Status 2 | -0.06376 | 0.05216 | 56000 | -1.22 | 0.2216 |
| Delay* Status 3 | -0.02802 | 0.05732 | 56000 | -0.49 | 0.6250 |
| Delay_cs* Status 1 | -0.02243 | 0.1078 | 56000 | -0.21 | 0.8351 |
| Delay_cs* Status 2 | 0.1954 | 0.1044 | 56000 | 1.87 | 0.0613 |
| Delay_cs* Status 3 | 0.1542 | 0.1187 | 56000 | 1.30 | 0.1942 |
| Beep number | -0.08818 | 0.02380 | 1525 | -3.70 | 0.0002 |
| Beep number_cs | 0.01268 | 0.02901 | 1526 | 0.44 | 0.6622 |
| Day number | -0.1907 | 0.01383 | 56000 | -13.78 | <.0001 |
| Random effects | β | *SE* | *Z* | *p* |  |
| Intercept | 0.3497 | 0.01657 | 21.10 | <.0001 |  |
| Delay | 0.08666 | 0.01720 | 5.04 | <.0001 |  |
| Delay_cs | 0.2424 | 0.07095 | 3.42 | 0.0003 |  |
| Beep number | 0.1695 | 0.03089 | 5.49 | <.0001 |  |
| Beep number_cs | 0.2734 | 0.04616 | 5.92 | <.0001 |  |
| Residual | 0.2957 | 0.001743 | 169.66 | <.0001 |  |
| *Notes. N* = 1528; Number of observations = 63863; Delay_cs = cubic spline transformed delay; Beep number_cs = cubic spline transformed beep number; Reference category = control, status 1 = individuals at risk for psychosis, status 2 = individuals with psychosis, status 3 = individuals with depression. | | | | | |

| Table S4. *The association between delay and positive affect with heterogeneous error variances. Coefficients for rescaled variables.* | | | | | |
| --- | --- | --- | --- | --- | --- |
| Fixed Effects | β | *SE* | *DF* | *t* | *p* |
| Intercept | 4.6376 | 0.03411 | 1523 | 135.97 | <.0001 |
| Delay | -0.1925 | 0.04670 | 1518 | -4.12 | <.0001 |
| Delay_cs | 0.2317 | 0.09861 | 1492 | 2.35 | 0.0189 |
| Status 1 | 0.07042 | 0.06910 | 57000 | 1.02 | 0.3081 |
| Status 2 | -0.4566 | 0.06677 | 57000 | -6.84 | <.0001 |
| Status 3 | -1.3018 | 0.07743 | 57000 | -16.81 | <.0001 |
| Delay* Status 1 | -0.01359 | 0.09295 | 57000 | -0.15 | 0.8838 |
| Delay* Status 2 | 0.1410 | 0.1027 | 57000 | 1.37 | 0.1697 |
| Delay* Status 3 | -0.03599 | 0.1163 | 57000 | -0.31 | 0.7569 |
| Delay_cs* Status 1 | 0.1579 | 0.1948 | 57000 | 0.81 | 0.4177 |
| Delay_cs* Status 2 | -0.2537 | 0.2050 | 57000 | -1.24 | 0.2160 |
| Delay_cs* Status 3 | 0.1438 | 0.2290 | 57000 | 0.63 | 0.5300 |
| Beep number | 0.6674 | 0.04758 | 1526 | 14.03 | <.0001 |
| Beep number_cs | -0.6251 | 0.05509 | 1527 | -11.35 | <.0001 |
| Day number | -0.09310 | 0.02498 | 57000 | -3.73 | 0.0002 |
| Random effects | β | *SE* | *Z* | *p* |  |
| Intercept | 0.9268 | 0.04611 | 20.10 | <.0001 |  |
| Delay | 0.4495 | 0.06418 | 7.00 | <.0001 |  |
| Delay_cs | 1.4398 | 0.2645 | 5.44 | <.0001 |  |
| Beep number | 1.1466 | 0.1250 | 9.17 | <.0001 |  |
| Beep number_cs | 1.3095 | 0.1678 | 7.80 | <.0001 |  |
| EXP status1 | -0.09278 | 0.02474 | -3.75 | 0.0002 |  |
| EXP status2 | 0.2310 | 0.02482 | 9.31 | <.0001 |  |
| EXP status3 | 0.4759 | 0.02615 | 18.20 | <.0001 |  |
| EXP delay | 0.02207 | 0.002060 | 10.71 | <.0001 |  |
| EXP status1*delay | -0.00556 | 0.004334 | -1.28 | 0.1993 |  |
| EXP status2*delay | -0.01271 | 0.003655 | -3.48 | 0.0005 |  |
| EXP status3*delay | -0.04130 | 0.004128 | -10.01 | <.0001 |  |
| Residual | 0.8436 | 0.009965 | 84.66 | <.0001 |  |
| Notes. *N* = 1528; Number of observations = 64110; Delay_cs = cubic spline transformed delay; Beep number_cs = cubic spline transformed beep number; Reference category = control, status 1 = individuals at risk for psychosis, status 2 = individuals with psychosis, status 3 = individuals with depression. | | | | | |

| Table S5.  *The association between delay and negative affect with heterogeneous error variances. Coefficients for rescaled variables.* | | | | | |
| --- | --- | --- | --- | --- | --- |
| Fixed Effects | β | *SE* | *DF* | *t* | *p* |
| Intercept | 1.3944 | 0.02061 | 1522 | 67.65 | <.0001 |
| Delay | 0.1417 | 0.02098 | 1517 | 6.76 | <.0001 |
| Delay_cs | -0.2180 | 0.04505 | 1491 | -4.84 | <.0001 |
| Status 1 | 0.1375 | 0.04313 | 56000 | 3.19 | 0.0014 |
| Status 2 | 0.5078 | 0.04198 | 56000 | 12.10 | <.0001 |
| Status 3 | 0.8663 | 0.04933 | 56000 | 17.56 | <.0001 |
| Delay* Status 1 | -0.00856 | 0.04401 | 56000 | -0.19 | 0.8457 |
| Delay* Status 2 | -0.07728 | 0.05557 | 56000 | -1.39 | 0.1643 |
| Delay* Status 3 | 0.01380 | 0.07247 | 56000 | 0.19 | 0.8490 |
| Delay_cs* Status 1 | 0.01222 | 0.09593 | 56000 | 0.13 | 0.8986 |
| Delay_cs* Status 2 | 0.2440 | 0.1157 | 56000 | 2.11 | 0.0349 |
| Delay_cs* Status 3 | 0.06603 | 0.1457 | 56000 | 0.45 | 0.6504 |
| Beep number | -0.07467 | 0.02135 | 1525 | -3.50 | 0.0005 |
| Beep number_cs | 0.01204 | 0.02617 | 1526 | 0.46 | 0.6457 |
| Day number | -0.1791 | 0.01262 | 56000 | -14.19 | <.0001 |
| Random effects | β | *SE* | *z* | *p* |  |
| Intercept | 0.3421 | 0.01653 | 20.70 | <.0001 |  |
| Delay | 0.07586 | 0.01482 | 5.12 | <.0001 |  |
| Delay_cs | 0.1647 | 0.06483 | 2.54 | 0.0055 |  |
| Beep number | 0.1228 | 0.02508 | 4.90 | <.0001 |  |
| Beep number_cs | 0.2097 | 0.03835 | 5.47 | <.0001 |  |
| EXP status1 | 0.1056 | 0.02489 | 4.24 | <.0001 |  |
| EXP status2 | 0.7639 | 0.02598 | 29.40 | <.0001 |  |
| EXP status3 | 1.4083 | 0.02590 | 54.38 | <.0001 |  |
| EXP delay | 0.04353 | 0.002200 | 19.79 | <.0001 |  |
| EXP status1*delay | 0.001225 | 0.004370 | 0.28 | 0.7793 |  |
| EXP status2*delay | -0.00687 | 0.003933 | -1.75 | 0.0806 |  |
| EXP status3*delay | -0.04621 | 0.004040 | -11.44 | <.0001 |  |
| Residual | 0.1622 | 0.001987 | 81.62 | <.0001 |  |
| *Notes. N* = 1528; Number of observations = 63863; Delay_cs = cubic spline transformed delay; Beep number_cs = cubic spline transformed beep number; Reference category = control, status 1 = individuals at risk for psychosis, status 2 = individuals with psychosis, status 3 = individuals with depression. | | | | | |

| Table S6. *The association between delay, positive affect, and activity stress. Coefficients for rescaled variables.* | | | | | |
| --- | --- | --- | --- | --- | --- |
| Fixed Effects | β | *SE* | *DF* | *t* | *p* |
| Intercept | 5.3198 | 0.04143 | 1438 | 128.40 | <.0001 |
| Delay | 0.06751 | 0.09941 | 1432 | 0.68 | 0.4972 |
| Delay_cs | -0.1347 | 0.2166 | 1404 | -0.62 | 0.5340 |
| Status 1 | -0.08092 | 0.08148 | 50000 | -0.99 | 0.3206 |
| Status 2 | -0.5134 | 0.08029 | 50000 | -6.39 | <.0001 |
| Status 3 | -0.7653 | 0.1028 | 50000 | -7.45 | <.0001 |
| Delay* Status 1 | -0.1409 | 0.1939 | 50000 | -0.73 | 0.4675 |
| Delay* Status 2 | -0.02847 | 0.2133 | 50000 | -0.13 | 0.8938 |
| Delay* Status 3 | -0.9459 | 0.2513 | 50000 | -3.76 | 0.0002 |
| Delay_cs* Status 1 | 0.1428 | 0.4269 | 50000 | 0.33 | 0.7380 |
| Delay_cs* Status 2 | -0.1289 | 0.4418 | 50000 | -0.29 | 0.7705 |
| Delay_cs* Status 3 | 2.1290 | 0.5761 | 50000 | 3.70 | 0.0002 |
| act_stress | -2.7773 | 0.1095 | 1437 | -25.36 | <.0001 |
| delay*act_stress | -0.9518 | 0.3585 | 50000 | -2.65 | 0.0079 |
| act_stress*status1 | 0.5045 | 0.2229 | 50000 | 2.26 | 0.0236 |
| act_stress*status2 | 0.4072 | 0.2140 | 50000 | 1.90 | 0.0571 |
| act_stress*status3 | 0.03969 | 0.2667 | 50000 | 0.15 | 0.8817 |
| delay*act_str*status | 0.3421 | 0.6898 | 50000 | 0.50 | 0.6199 |
| delay*act_str*status | 0.6896 | 0.7268 | 50000 | 0.95 | 0.3427 |
| delay*act_str*status | 3.4506 | 0.8318 | 50000 | 4.15 | <.0001 |
| act_stress*delay_cs | 1.3504 | 0.7803 | 50000 | 1.73 | 0.0835 |
| act_st*delay_cs*status | 0.5243 | 1.5385 | 50000 | 0.34 | 0.7333 |
| act_st*delay_cs*status | -0.4665 | 1.4979 | 50000 | -0.31 | 0.7555 |
| act_st*delay_cs*status | -6.9298 | 1.9221 | 50000 | -3.61 | 0.0003 |
| Beep number | 0.7883 | 0.04956 | 1440 | 15.91 | <.0001 |
| Beep number_cs | -0.8433 | 0.05658 | 1440 | -14.90 | <.0001 |
| Day number | -0.07731 | 0.02596 | 50000 | -2.98 | 0.0029 |
| Random effects | β | *SE* | *z* | *p* |  |
| Intercept | 0.9559 | 0.05561 | 17.19 | <.0001 |  |
| Delay | 0.3955 | 0.06434 | 6.15 | <.0001 |  |
| Delay_cs | 1.3458 | 0.2679 | 5.02 | <.0001 |  |
| Beep number | 1.2037 | 0.1304 | 9.23 | <.0001 |  |
| Beep number_cs | 1.2457 | 0.1706 | 7.30 | <.0001 |  |
| Act_stress | 2.8875 | 0.2186 | 13.21 | <.0001 |  |
| Residual | 0.9172 | 0.005732 | 160.00 | <.0001 |  |
| Notes. *N* = 1442; Number of observations = 58253; Delay_cs = cubic spline transformed delay; Beep number_cs = cubic spline transformed beep number; Reference category = control, status 1 = individuals at risk for psychosis, status 2 = individuals with psychosis, status 3 = individuals with depression. | | | | | |

| Table S7. *The association between delay, positive affect, and event stress. Coefficients for rescaled variables.* | | | | | |
| --- | --- | --- | --- | --- | --- |
| Fixed Effects | β | *SE* | *DF* | *t* | *p* |
| Intercept | 5.1664 | 0.03733 | 1432 | 138.39 | <.0001 |
| Delay | -0.00281 | 0.08420 | 1415 | -0.03 | 0.9734 |
| Delay_cs | 0.05304 | 0.1857 | 1371 | 0.29 | 0.7752 |
| Status 1 | 0.04364 | 0.07069 | 44000 | 0.62 | 0.5370 |
| Status 2 | -0.4468 | 0.06714 | 44000 | -6.65 | <.0001 |
| Status 3 | -0.5966 | 0.08766 | 44000 | -6.81 | <.0001 |
| Delay* Status 1 | 0.01240 | 0.1555 | 44000 | 0.08 | 0.9364 |
| Delay* Status 2 | 0.06965 | 0.1601 | 44000 | 0.44 | 0.6635 |
| Delay* Status 3 | -0.3338 | 0.1910 | 44000 | -1.75 | 0.0806 |
| Delay_cs* Status 1 | -0.2386 | 0.3465 | 44000 | -0.69 | 0.4911 |
| Delay_cs* Status 2 | -0.3030 | 0.3329 | 44000 | -0.91 | 0.3627 |
| Delay_cs* Status 3 | 0.7148 | 0.4327 | 44000 | 1.65 | 0.0985 |
| event_stress | -1.6395 | 0.07047 | 1393 | -23.27 | <.0001 |
| delay*event_stress | -0.6627 | 0.2457 | 44000 | -2.70 | 0.0070 |
| event_stress*status1 | -0.07477 | 0.1392 | 44000 | -0.54 | 0.5913 |
| event_stress*status2 | -0.1372 | 0.1290 | 44000 | -1.06 | 0.2873 |
| event_stress*status3 | -0.9217 | 0.1623 | 44000 | -5.68 | <.0001 |
| delay*event_str*status | -0.05854 | 0.4609 | 44000 | -0.13 | 0.8989 |
| delay*event_str*status | 0.3081 | 0.4628 | 44000 | 0.67 | 0.5056 |
| delay*event_str*status | 1.0627 | 0.5298 | 44000 | 2.01 | 0.0449 |
| event_stress*delay_cs | 0.5979 | 0.5555 | 44000 | 1.08 | 0.2818 |
| event_st*delay_cs*status | 1.2729 | 1.0562 | 44000 | 1.21 | 0.2281 |
| event_st*delay_cs*status | 0.2074 | 0.9719 | 44000 | 0.21 | 0.8311 |
| event_st*delay_cs*status | -1.6058 | 1.2003 | 44000 | -1.34 | 0.1810 |
| Beep number | 0.5044 | 0.05015 | 1419 | 10.06 | <.0001 |
| Beep number_cs | -0.5272 | 0.05818 | 1411 | -9.06 | <.0001 |
| Day number | -0.07122 | 0.02701 | 44000 | -2.64 | 0.0084 |
| Random effects | β | *SE* | *Z* | *p* |  |
| Intercept | 0.7638 | 0.04750 | 16.08 | <.0001 |  |
| Delay | 0.3810 | 0.06815 | 5.59 | <.0001 |  |
| Delay_cs | 1.3514 | 0.2880 | 4.69 | <.0001 |  |
| Beep number | 0.9598 | 0.1312 | 7.32 | <.0001 |  |
| Beep number_cs | 1.0871 | 0.1778 | 6.11 | <.0001 |  |
| Event_stress | 0.8941 | 0.07930 | 11.28 | <.0001 |  |
| Residual | 0.9226 | 0.006074 | 151.90 | <.0001 |  |
| Notes. *N* = 1437; Number of observations = 52946;Delay_cs = cubic spline transformed delay; Beep number_cs = cubic spline transformed beep number; Reference category = control, status 1 = individuals at risk for psychosis, status 2 = individuals with psychosis, status 3 = individuals with depression. | | | | | |

| Table S8. *The association between delay, negative affect, and activity stress. Coefficients for rescaled variables.* | | | | | |
| --- | --- | --- | --- | --- | --- |
| Fixed Effects | β | *SE* | *DF* | *t* | *p* |
| Intercept | 1.1696 | 0.02112 | 1438 | 55.37 | <.0001 |
| Delay | -0.00746 | 0.05305 | 1432 | -0.14 | 0.8882 |
| Delay_cs | -0.01486 | 0.1170 | 1404 | -0.13 | 0.8990 |
| Status 1 | 0.1040 | 0.04152 | 49000 | 2.50 | 0.0123 |
| Status 2 | 0.3081 | 0.04106 | 49000 | 7.50 | <.0001 |
| Status 3 | 0.4693 | 0.05230 | 49000 | 8.97 | <.0001 |
| Delay* Status 1 | -0.07448 | 0.1035 | 49000 | -0.72 | 0.4717 |
| Delay* Status 2 | 0.05724 | 0.1131 | 49000 | 0.51 | 0.6127 |
| Delay* Status 3 | 0.6869 | 0.1341 | 49000 | 5.12 | <.0001 |
| Delay_cs* Status 1 | 0.05720 | 0.2308 | 49000 | 0.25 | 0.8043 |
| Delay_cs* Status 2 | 0.1363 | 0.2367 | 49000 | 0.58 | 0.5647 |
| Delay_cs* Status 3 | -1.5208 | 0.3136 | 49000 | -4.85 | <.0001 |
| act_stress | 0.9907 | 0.06548 | 1437 | 15.13 | <.0001 |
| delay*act_stress | 0.6421 | 0.1959 | 49000 | 3.28 | 0.0010 |
| act_stress*status1 | 0.1048 | 0.1338 | 49000 | 0.78 | 0.4332 |
| act_stress*status2 | 0.5695 | 0.1287 | 49000 | 4.42 | <.0001 |
| act_stress*status3 | 0.8743 | 0.1618 | 49000 | 5.41 | <.0001 |
| delay*act_str*status | 0.3080 | 0.3764 | 49000 | 0.82 | 0.4133 |
| delay*act_str*status | -0.5830 | 0.3925 | 49000 | -1.49 | 0.1374 |
| delay*act_str*status | -2.7294 | 0.4554 | 49000 | -5.99 | <.0001 |
| act_stress*delay_cs | -0.9297 | 0.4297 | 49000 | -2.16 | 0.0305 |
| act_st*delay_cs*status | -0.3724 | 0.8448 | 49000 | -0.44 | 0.6593 |
| act_st*delay_cs*status | 0.4167 | 0.8139 | 49000 | 0.51 | 0.6087 |
| act_st*delay_cs*status | 5.9384 | 1.0650 | 49000 | 5.58 | <.0001 |
| Beep number | -0.1593 | 0.02407 | 1440 | -6.62 | <.0001 |
| Beep number_cs | 0.1362 | 0.02881 | 1440 | 4.73 | <.0001 |
| Day number | -0.2001 | 0.01407 | 49000 | -14.22 | <.0001 |
| Random effects | β | *SE* | *Z* | *p* |  |
| Intercept | 0.2137 | 0.01397 | 15.31 | <.0001 |  |
| Delay | 0.08585 | 0.01693 | 5.07 | <.0001 |  |
| Delay_cs | 0.2814 | 0.07215 | 3.90 | <.0001 |  |
| Beep number | 0.1625 | 0.03105 | 5.24 | <.0001 |  |
| Beep number_cs | 0.2168 | 0.04505 | 4.81 | <.0001 |  |
| Act_stress | 1.4541 | 0.08613 | 16.88 | <.0001 |  |
| Residual | 0.2686 | 0.001683 | 159.59 | <.0001 |  |
| Notes. *N* = 1442; Number of observations = 58050; Delay_cs = cubic spline transformed delay; Beep number_cs = cubic spline transformed beep number; Reference category = control, status 1 = individuals at risk for psychosis, status 2 = individuals with psychosis, status 3 = individuals with depression. | | | | | |

| Table S9. *The association between delay, negative affect, and event stress. Coefficients for rescaled variables.* | | | | | |
| --- | --- | --- | --- | --- | --- |
| Fixed Effects | β | *SE* | *DF* | *t* | *p* |
| Intercept | 1.2148 | 0.02165 | 1431 | 56.11 | <.0001 |
| Delay | 0.01655 | 0.04572 | 1414 | 0.36 | 0.7174 |
| Delay_cs | -0.05967 | 0.09985 | 1370 | -0.60 | 0.5502 |
| Status 1 | 0.09365 | 0.04145 | 44000 | 2.26 | 0.0239 |
| Status 2 | 0.4539 | 0.03946 | 44000 | 11.50 | <.0001 |
| Status 3 | 0.5194 | 0.05158 | 44000 | 10.07 | <.0001 |
| Delay* Status 1 | -0.05822 | 0.08411 | 44000 | -0.69 | 0.4888 |
| Delay* Status 2 | -0.07518 | 0.08631 | 44000 | -0.87 | 0.3837 |
| Delay* Status 3 | 0.2947 | 0.1034 | 44000 | 2.85 | 0.0044 |
| Delay_cs* Status 1 | 0.3046 | 0.1847 | 44000 | 1.65 | 0.0992 |
| Delay_cs* Status 2 | 0.2326 | 0.1769 | 44000 | 1.32 | 0.1885 |
| Delay_cs* Status 3 | -0.4145 | 0.2316 | 44000 | -1.79 | 0.0735 |
| event_stress | 0.6246 | 0.04272 | 1392 | 14.62 | <.0001 |
| delay*event_stress | 0.4088 | 0.1382 | 44000 | 2.96 | 0.0031 |
| event_stress*status1 | 0.1705 | 0.08513 | 44000 | 2.00 | 0.0452 |
| event_stress*status2 | 0.1607 | 0.07965 | 44000 | 2.02 | 0.0437 |
| event_stress*status3 | 0.8194 | 0.1003 | 44000 | 8.17 | <.0001 |
| delay*event_str*status | 0.2614 | 0.2592 | 44000 | 1.01 | 0.3132 |
| delay*event_str*status | 0.05739 | 0.2579 | 44000 | 0.22 | 0.8239 |
| delay*event_str*status | -1.1612 | 0.2975 | 44000 | -3.90 | <.0001 |
| event_stress*delay_cs | -0.4720 | 0.3081 | 44000 | -1.53 | 0.1255 |
| event_st*delay_cs*status | -1.2218 | 0.5836 | 44000 | -2.09 | 0.0363 |
| event_st*delay_cs*status | -0.2626 | 0.5327 | 44000 | -0.49 | 0.6220 |
| event_st*delay_cs*status | 1.7740 | 0.6626 | 44000 | 2.68 | 0.0074 |
| Beep number | -0.04236 | 0.02516 | 1419 | -1.68 | 0.0925 |
| Beep number_cs | 0.002983 | 0.02979 | 1410 | 0.10 | 0.9202 |
| Day number | -0.1959 | 0.01486 | 44000 | -13.19 | <.0001 |
| Random effects | β | *SE* | *Z* | *p* |  |
| Intercept | 0.2655 | 0.01572 | 16.89 | <.0001 |  |
| Delay | 0.07446 | 0.01788 | 4.17 | <.0001 |  |
| Delay_cs | 0.1506 | 0.07300 | 2.06 | 0.0195 |  |
| Beep number | 0.1285 | 0.03281 | 3.92 | <.0001 |  |
| Beep number_cs | 0.1551 | 0.04621 | 3.36 | 0.0004 |  |
| Event_stress | 0.4933 | 0.03304 | 14.93 | <.0001 |  |
| Residual | 0.2780 | 0.001834 | 151.56 | <.0001 |  |
| Notes. *N* = 1437; Number of observations = 52748; Delay_cs = cubic spline transformed delay; Beep number_cs = cubic spline transformed beep number; Reference category = control, status 1 = individuals at risk for psychosis, status 2 = individuals with psychosis, status 3 = individuals with depression. | | | | | |

**Code Analyses**

R Code

# Preprocessing: Rescaling, centering, inverse coding & calculating mean scores.

dat$delay <- (dat$delay / 10)

dat$delay <- (dat$delay - 0.5)

dat$beepno <- (dat$beepno - 1)

dat$beepno <- (dat$beepno / 10)

dat$dayno <- (dat$dayno - 1)

dat$dayno <- (dat$dayno / 10)

dat$act_well_inversed <- 8 - dat$act_well

dat$eve_pleasant_inversed <- 8 - (dat$eve_pleasant + 4)

dat$act_stress <- (dat$act_well_inversed + dat$act_challeng + dat$act_difficul + dat$act_else) / 4

dat$act_stress <- dat$act_stress / 10

dat$event_stress <- dat$eve_pleasant_inversed / 10

# Cubic spline transformation

deltime_knots <- c(-0.2, 0.0, 0.7)

dat$delay_cs <- c(rcspline.eval(dat$delay,deltime_knots))

beepno_knots <- c(0.19, 0.55, 0.91)

dat$beepno_cs <- c(rcspline.eval(dat$beepno, beepno_knots))

# Research question 1: Changes in mean levels of affect as a function of delay pa <-

lme(

fixed = pa ~ delay * status + delay_cs * status + beepno + beepno_cs +

dayno,

random = ~ delay + delay_cs + beepno + beepno_cs |

subjno,

data = dat,

na.action = na.exclude,

control = list(opt = "optim"),

method = "ML"

)

na <-

lme(

fixed = na ~ delay * status + delay_cs * status + beepno + beepno_cs +

dayno ,

random = ~ delay_cs + delay + beepno + beepno_cs |

subjno,

data = dat,

na.action = na.exclude,

control = list(opt = "optim"),

method = "ML"

)

#Research question 2: Changes in within-person error variances of affect as a function of delay

pa.weights <-

lme(

pa ~ delay * status + delay_cs * status + beepno + beepno_cs +

dayno,

random = ~ delay + delay_cs + beepno + beepno_cs | subjno,

data = dat,

na.action = na.omit,

weights = varComb(varIdent(form = ~ 1 |

status), varExp(form = ~ deltime)),

control = list(opt = "optim", msVerbose = TRUE),

method = "ML"

)

na.weights <-

lme(

na ~ delay * status + delay_cs * status + beepno + beepno_cs +

dayno,

random = ~ delay + delay_cs + beepno + beepno_cs | subjno,

data = dat,

na.action = na.omit,

weights = varComb(varIdent(form = ~ 1 |

status), varExp(form = ~ deltime |

status)),

control = list(

opt = "nlminb",

maxIter = 5000,

msMaxIter = 5000,

msMaxEval = 5000,

msVerbose = TRUE

),

method = "ML"

)

# Research question 3: Changes in associations between affect and contextual stress variables as a function #of delay

####PA event_stress####

pa.event <-

lme(

fixed = pa ~ delay * status * event_stress + delay_cs * status * event_stress +

beepno + beepno_cs + dayno ,

random = ~ delay + delay_cs + beepno + beepno_cs +

event_stress |

subjno,

data = dat,

na.action = na.exclude,

control = list(opt = "optim"),

method = "ML"

)

###PA activity stress####

pa.act <-

lme(

fixed = pa ~ delay * status * act_stress + delay_cs * status * act_stress +

beepno + beepno_cs + dayno ,

random = ~ delay + delay_cs + beepno + beepno_cs +

act_stress |

subjno,

data = dat,

na.action = na.exclude,

control = list(opt = "optim"),

method = "ML"

)

##NA event_stress##########

#full model

na.event <-

lme(

fixed = na ~ delay * status * event_stress + delay_cs * status * event_stress +

beepno + beepno_cs + dayno,

random = ~ delay + delay_cs + beepno + beepno_cs +

event_stress |

subjno,

data = dat,

na.action = na.exclude,

control = list(opt = "optim"),

method = "ML"

)

###NA activity stress####

na.act <-

lme(

fixed = na ~ delay * status * act_stress + delay_cs * status * act_stress +

beepno + beepno_cs + dayno ,

random = ~ delay + delay_cs + beepno + beepno_cs +

act_stress |

subjno,

data = dat,

na.action = na.exclude,

control = list(opt = "optim"),

method = "ML"

)

SAS Code

* Research question 1: Changes in mean levels of affect as a function of delay;

proc mixed data=dat method=ml covtest;

class subjno status(ref="0");

model pa=delay|status delay_cs|status beepno beepno_cs dayno / solution;

random int delay delay_cs beepno beepno_cs / sub=subjno type=un;

contrast 'status0' delay 1 delay*status 0 0 0 1, delay_cs 1 delay_cs*status 0

0 0 1/Chisq e;

contrast 'interaction with status' delay*status 1 0 0 -1, delay*status 0 1

0 -1, delay*status 0 0 1 -1, delay_cs*status 1 0 0 -1, delay_cs*status 0 1

0 -1, delay_cs*status 0 0 1 -1/ CHISQ e;

run;

proc mixed data=dat method=ml covtest;

class subjno status(ref="0");

model na=delay|status delay_cs|status beepno beepno_cs dayno / solution;

random int delay delay_cs beepno beepno_cs / sub=subjno type=un;

contrast 'status0' delay 1 delay*status 0 0 0 1, delay_cs 1 delay_cs*status 0

0 0 1/Chisq e;

contrast 'interaction with status' delay*status 1 0 0 -1, delay*status 0 1

0 -1, delay*status 0 0 1 -1, delay_cs*status 1 0 0 -1, delay_cs*status 0 1

0 -1, delay_cs*status 0 0 1 -1/ CHISQ e;

run;

* Research question 2: Changes in within-person error variances of affect as a function of delay;

proc mixed data=dat method=ml covtest;

class subjno status(ref="0");

model pa=delay delay_cs status delay*status delay_cs*status beepno beepno_cs

dayno / solution;

random int delay delay_cs beepno beepno_cs / sub=subjno type=un;

ods output CovParms=cov;

repeated / local=exp(status1 status2 status3 deltime deltime*status1

deltime*status2 deltime*status3);

run;

* model for na does only converge when different starting values are added to the code;

proc mixed data=dat method=ml covtest;

class subjno status(ref="0");

model na=delay delay_cs status delay*status delay_cs*status beepno beepno_cs

dayno / solution;

random int delay delay_cs beepno beepno_cs / sub=subjno type=un;

ods output CovParms=cov;

repeated / local=exp(status1 status2 status3 deltime deltime*status1

deltime*status2 deltime*status3);

parms (0.34212572) (0.04336848) (0.07586137) (-0.08119909) (-0.11829157)

(0.16466817) (-0.04412924) (0.01827711) (-0.07144620) (0.12284330)

(0.02273706) (-0.03831947) (0.11497314) (-0.12767409) (0.20970308)

(0.10563977) (0.76385985) (1.40834561) (0.04353252) (0.04475769) (0.03666070)

(-0.00267508) (0.16219097);

run;

* Research question 3: Changes in associations between affect and contextual stress variables as a function of delay;

*Pa act_stress;

proc mixed data=dat method=ml covtest;

class subjno status(Ref="0");

model pa=delay|status|act_stress delay_cs|status|act_stress beepno beepno_cs

dayno / solution;

random int delay delay_cs beepno beepno_cs act_stress / sub=subjno type=un;

contrast 'delay*act_stress control' delay*act_stress 1 delay*act_stress*status

0 0 0 1, delay_cs*act_stress 1 delay_cs*act_stress* status 0 0 0 1 / CHISQ e;

contrast 'delay*act_stress*Status' delay*act_stress* status 1 0 0 -1,

delay*act_stress* status 0 1 0 -1, delay*act_stress* status 0 0 1 -1,

delay_cs*act_stress* status 1 0 0 -1, delay_cs*act_stress* status 0 1 0 -1,

delay_cs*act_stress* status 0 0 1 -1/ CHISQ e;

contrast 'at risk' delay*act_stress* status 1 0 0 -1, delay_cs*act_stress*

status 1 0 0 -1/ CHISQ e;

contrast 'psychotic' delay*act_stress* status 0 1 0 -1, delay_cs*act_stress*

status 0 1 0 -1/ CHISQ e;

contrast 'depressed' delay*act_stress* status 0 0 1 -1, delay_cs*act_stress*

status 0 0 1 -1/ CHISQ e;

run;

*Pa event_stress;

proc mixed data=dat method=ml covtest;

class subjno status(Ref="0");

model pa=delay|status|event_stress delay_cs|status|event_stress beepno

beepno_cs dayno / solution;

random int delay delay_cs beepno beepno_cs event_stress / sub=subjno type=un;

contrast 'delay*event_stress control' delay*event_stress 1

delay*event_stress*status 0 0 0 1, delay_cs*event_stress 1

delay_cs*event_stress* status 0 0 0 1 / CHISQ e;

contrast 'delay*event_stress*Status' delay*event_stress* status 1 0 0 -1,

delay*event_stress* status 0 1 0 -1, delay*event_stress* status 0 0 1 -1,

delay_cs*event_stress* status 1 0 0 -1, delay_cs*event_stress* status 0 1

0 -1, delay_cs*event_stress* status 0 0 1 -1/ CHISQ e;

contrast 'at risk' delay*event_stress* status 1 0 0 -1, delay_cs*event_stress*

status 1 0 0 -1/ CHISQ e;

contrast 'psychotic' delay*event_stress* status 0 1 0 -1,

delay_cs*event_stress* status 0 1 0 -1/ CHISQ e;

contrast 'depressed' delay*event_stress* status 0 0 1 -1,

delay_cs*event_stress* status 0 0 1 -1/ CHISQ e;

run;

*Na act_stress;

proc mixed data=dat method=ml covtest;

class subjno status(Ref="0");

model na=delay|status|act_stress delay_cs|status|act_stress beepno beepno_cs

dayno / solution;

random int delay delay_cs beepno beepno_cs act_stress / sub=subjno type=un;

contrast 'delay*act_stress control' delay*act_stress 1 delay*act_stress*status

0 0 0 1, delay_cs*act_stress 1 delay_cs*act_stress* status 0 0 0 1 / CHISQ e;

contrast 'delay*act_stress*Status' delay*act_stress* status 1 0 0 -1,

delay*act_stress* status 0 1 0 -1, delay*act_stress* status 0 0 1 -1,

delay_cs*act_stress* status 1 0 0 -1, delay_cs*act_stress* status 0 1 0 -1,

delay_cs*act_stress* status 0 0 1 -1/ CHISQ e;

contrast 'at risk' delay*act_stress* status 1 0 0 -1, delay_cs*act_stress*

status 1 0 0 -1/ CHISQ e;

contrast 'psychotic' delay*act_stress* status 0 1 0 -1, delay_cs*act_stress*

status 0 1 0 -1/ CHISQ e;

contrast 'depressed' delay*act_stress* status 0 0 1 -1, delay_cs*act_stress*

status 0 0 1 -1/ CHISQ e;

run;

*Na event_stress;

proc mixed data=dat method=ml covtest;

class subjno status(Ref="0");

model na=delay|status|event_stress delay_cs|status|event_stress beepno

beepno_cs dayno / solution;

random int delay delay_cs beepno beepno_cs event_stress / sub=subjno type=un;

contrast 'delay*event_stress control' delay*event_stress 1

delay*event_stress*status 0 0 0 1, delay_cs*event_stress 1

delay_cs*event_stress* status 0 0 0 1 / CHISQ e;

contrast 'delay*event_stress*Status' delay*event_stress* status 1 0 0 -1,

delay*event_stress* status 0 1 0 -1, delay*event_stress* status 0 0 1 -1,

delay_cs*event_stress* status 1 0 0 -1, delay_cs*event_stress* status 0 1

0 -1, delay_cs*event_stress* status 0 0 1 -1/ CHISQ e;

contrast 'at risk' delay*event_stress* status 1 0 0 -1, delay_cs*event_stress*

status 1 0 0 -1/ CHISQ e;

contrast 'psychotic' delay*event_stress* status 0 1 0 -1,

delay_cs*event_stress* status 0 1 0 -1/ CHISQ e;

contrast 'depressed' delay*event_stress* status 0 0 1 -1,

delay_cs*event_stress* status 0 0 1 -1/ CHISQ e;

run;
